# Vagal nerve stimulation improves mitochondrial dynamics *via* an M_3_ receptor/CaMKKβ/AMPK pathway in isoproterenol‐induced myocardial ischaemia

**DOI:** 10.1111/jcmm.12938

**Published:** 2016-08-05

**Authors:** Run‐Qing Xue, Lei Sun, Xiao‐Jiang Yu, Dong‐Ling Li, Wei‐Jin Zang

**Affiliations:** ^1^ Department of PharmacologySchool of Basic Medical SciencesXi'an Jiaotong University Health Science CenterXi'anChina

**Keywords:** myocardial ischaemia, vagal nerve stimulation, mitochondrial dynamics, mitochondrial function, AMP‐activated protein kinase, subtype‐3 of muscarinic acetylcholine receptor, cardioprotection

## Abstract

Mitochondrial dynamics—fission and fusion—are associated with ischaemic heart disease (IHD). This study explored the protective effect of vagal nerve stimulation (VNS) against isoproterenol (ISO)‐induced myocardial ischaemia in a rat model and tested whether VNS plays a role in preventing disorders of mitochondrial dynamics and function. Isoproterenol not only caused cardiac injury but also increased the expression of mitochondrial fission proteins [dynamin‐related peptide1 (Drp1) and mitochondrial fission protein1 (Fis‐1)) and decreased the expression of fusion proteins (optic atrophy‐1 (OPA1) and mitofusins1/2 (Mfn1/2)], thereby disrupting mitochondrial dynamics and leading to increase in mitochondrial fragments. Interestingly, VNS restored mitochondrial dynamics through regulation of Drp1, Fis‐1, OPA1 and Mfn1/2; enhanced ATP content and mitochondrial membrane potential; reduced mitochondrial permeability transition pore (MPTP) opening; and improved mitochondrial ultrastructure and size. Furthermore, VNS reduced the size of the myocardial infarction and ameliorated cardiomyocyte apoptosis and cardiac dysfunction induced by ISO. Moreover, VNS activated AMP‐activated protein kinase (AMPK), which was accompanied by phosphorylation of Ca^2+^/calmodulin‐dependent protein kinase kinase β (CaMKKβ) during myocardial ischaemia. Treatment with subtype‐3 of muscarinic acetylcholine receptor (M_3_R) antagonist 4‐diphenylacetoxy‐*N*‐methylpiperidine methiodide or AMPK inhibitor Compound C abolished the protective effects of VNS on mitochondrial dynamics and function, suggesting that M_3_R/CaMKKβ/AMPK signalling are involved in mediating beneficial effects of VNS. This study demonstrates that VNS modulates mitochondrial dynamics and improves mitochondrial function, possibly through the M_3_R/CaMKKβ/AMPK pathway, to attenuate ISO‐induced cardiac damage in rats. Targeting mitochondrial dynamics may provide a novel therapeutic strategy in IHD.

## Introduction

In ischaemic heart disease (IHD), which continues to be the main cause of the death worldwide, heart mitochondria directly sustain injury 1. Isoproterenol (ISO)‐induced myocardial ischaemia induces myocardial damage similar to that in patients with myocardial infarction (MI) and is commonly used for generating an experimental model in rats 2, 3. Injury to mitochondrial ultrastructure and function occurs during early ischaemia and progresses during sustained ischaemia. Mitochondrial dysfunction plays a major role in myocardial ischaemia 4, with phenotypes including decreased mitochondrial metabolic enzymes and ATP content and opening of the mitochondrial permeability transition pore (MPTP), which results in a burst of reactive oxygen species (ROS) and Ca^2+^ uptake, leading to apoptosis or necrosis 5, 6. Importantly, mitochondrial function relies heavily on changes to mitochondrial ultrastructure and morphology—the phenomenon of mitochondrial dynamics 7. Dynamic mitochondria constantly undergo fusion and fission, with these two opposing processes regulated by mitochondrial fusion [optic atrophy‐1 (OPA1), and mitofusins1 and 2 (Mfn1/2)] and fission proteins [dynamin‐related peptide1 (Drp1), and mitochondrial fission protein1 (Fis‐1)] respectively 8. Both mitochondrial fission and fusion are essential for cell metabolic function and facilitate segregation of dysfunctional or damaged mitochondria before apoptosis 9, 10. Regulation of proteins mediating mitochondrial dynamics or inhibition of excessive mitochondrial fission attenuates mitochondrial dysfunction to improve MI 11, 12. Therefore, targeting these proteins that regulate mitochondrial dynamics could prevent cardiac injury occurring due to myocardial ischaemia.

Our previous research showed that acetylcholine (ACh), the major neurotransmitter of the vagal nerve, inhibits ROS formation, improves mitochondrial biogenesis and initiates a mitophagy process to mitigate myocardial ischaemia–reperfusion injury (IRI) 13, 14, 15, 16. Interestingly, clinical studies have reported that imbalances in the cardiac autonomic nervous system, especially reduced vagal activity, are relevant to the pathogenesis of IHD 17, thus bringing increased focus on enhancing vagal activity as a potential therapeutic option to cope with IHD 18. Moreover, previous studies have shown that enhanced vagal activity has a positive effect of reducing injury and enhancing recovery of myocardial function in both animal studies and clinical practice 19, 20, 21. Cumulative studies have shown vagal nerve stimulation (VNS) prevents both myocardial ischaemia and burn injury through attenuation of mitochondrial dysfunction and suppression of myocardial apoptosis 22, 23. Although VNS and ACh have beneficial effects on mitochondria that, in turn, have a cardioprotective role, the mechanism by which VNS regulates mitochondrial dynamics following myocardial ischaemia is not fully understood.

AMP‐activated protein kinase (AMPK), a key cellular energy sensor and regulator of metabolic homoeostasis, modulates mitochondrial function, endoplasmic reticulum (ER) stress, autophagy and apoptosis, and prevents myocardial necrosis and contractile dysfunction during MI 24. Furthermore, a previous study from our laboratory showed ACh promotes cell survival *via* an AMPK‐induced cardiomyocyte autophagy pathway during cardiomyocyte hypoxia/reoxygenation injury 13. Increasing evidence suggests AMPK acts as a hub to bridge mitochondrial dysfunction and IHD 25, 26; however, AMPK's role in mitochondrial dynamics regulation during myocardial ischaemia remains unknown. This study aimed to elucidate this role using ISO‐induced myocardial ischaemia in a rat model by examining the protective effects of VNS on mitochondrial dynamics and function, with specific focus on AMPK‐related pathways.

## Materials and methods

### Animals and induction of myocardial ischaemia by ISO

Male Sprague–Dawley (SD; 180–200 g) rats were obtained from Xi'an Jiaotong University Laboratorial Animal Center and housed under standard conditions, with aces to food and water *ad libitum*. Isoproterenol 25 mg/kg (Sigma‐Aldrich, Saint Louis, MO, USA) dissolved in saline was administered through subcutaneous injections to the rats at 24‐hr intervals for 2 days to induce myocardial injury, on the basis of preliminary experimental results discussed previously 27, 28. All experiments in this study conformed to the Guideline on the Care and Use of Laboratory Animals and were approved by the Ethics Committee of Xi'an Jiaotong University.

### Vagal nerve stimulation

With subjects under general anaesthesia and mechanical ventilation, the right cervical vagal nerve was identified and transected in the neck region. A pair of platinum wires was placed at the distal end of the vagal nerve for stimulation, and the electrode was connected to an isolated constant voltage stimulator (Power Lab; AD Instruments, Bella Vista, New South Wales, Australia). The vagal nerve was stimulated with electrical rectangular pulses of 2 Hz for 1 msec. Electrical voltage pulses ranging from 2 to 4 V were required to obtain a 10% reduction in basal heart rate (HR) 21. Vagal nerve stimulation was performed 60 min. after the last ISO administration and was continued for 240 min.

### Animal experiment protocol

**Figure 1:**
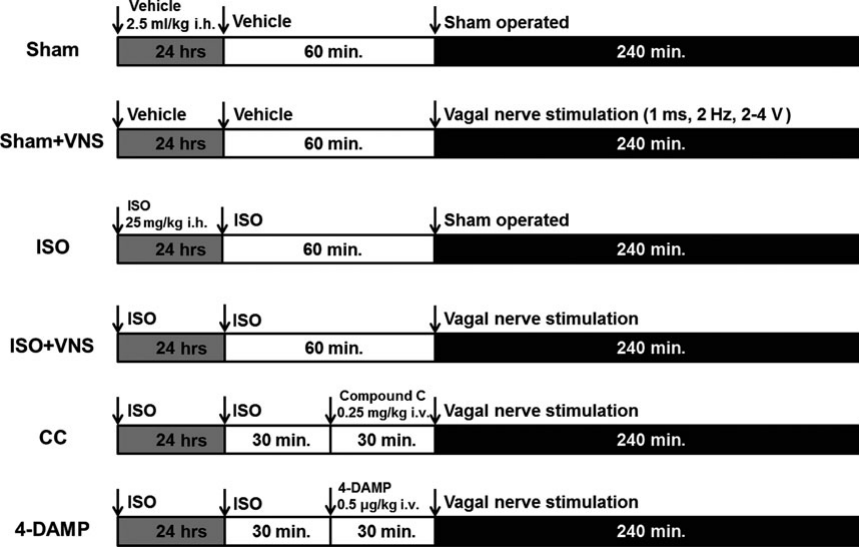
Experimental protocol. Sham, vehicle‐treated without vagal nerve stimulation. Sham+VNS: vehicle‐treated with vagal nerve stimulation; ISO, ISO‐treated without vagal nerve stimulation. ISO+VNS, ISO‐treated with vagal nerve stimulation. CC (ISO+VNS+Compound C), the rats were treated with Compound C (0.25 mg/kg, IV) 30 min. prior to VNS treatment. 4‐DAMP (ISO+VNS+4‐DAMP), 4‐DAMP (0.5 μg/kg, IV) was injected 30 min. prior to VNS treatment.

Sprague–Dawley rats were randomized into four groups: (*i*) vehicle‐treated without VNS (Sham), (*ii*) vehicle‐treated with VNS (Sham+VNS), (*iii*) ISO‐treated without VNS (ISO) and (*iv*) ISO‐treated with VNS (ISO+VNS). Separate sets of rats were studied for *in vivo* hemodynamics prior to sacrifice and their tissue samples were subject to electron microscope morphological analysis and protein assays (*n* = 6). Another set of rats was killed after experimental operation for the study of isolated heart mitochondria (*n* = 6). To examine the effects of the AMPK‐related signalling pathway, another set of rats were administered Compound C (an AMPK inhibitor; Sigma‐Aldrich, 0.25 mg/kg bw through tail vein injection 30 min. before VNS) and 4‐diphenylacetoxy‐*N*‐methylpiperidine methiodide (4‐DAMP, a selective subtype‐3 of a muscarinic acetylcholine receptor (M_3_R) antagonist, Sigma‐Aldrich; 0.5 μg/kg bw through tail vein injection 30 min. before VNS) 29. Treatment with Compound C or 4‐DAMP at doses specified did not significantly influence physiological parameters (data not shown). The experimental protocol and study animal disposition is presented in Figure 1.

### Hemodynamic measurements

Hemodynamic parameters were assessed by invasive LV catheterization and recorded using a polygraph recorder (PowerLab; AD Instruments) as described previously. Briefly, the left ventricle was catheterized with a heparin‐filled polyethylene catheter from the right common carotid artery to measure LV end‐diastolic pressure (LVEDP) and maximal rate of increase/decrease in LV pressure (±dP/dt_max_). The right femoral artery was catheterized and connected to a transducer for recording blood pressure and HR during the experiment. In addition, needle electrodes were inserted subcutaneously for the limb lead at position II for electrocardiographic monitoring.

### Measurement of myocardial infarct size

Following the experiment, the heart was excised immediately and sliced transversely into 2–3 mm‐thick sections, which were incubated in 1% 2,3,5‐triphenyltetrazolium chloride (Sigma‐Aldrich) solution for 30 min. at 37°C in the dark, fixed in 10% formalin, and photographed using a digital camera. The non‐infarcted myocardium was stained bright red, whereas infarcted myocardium appeared pale grey. Infarct size percentage was calculated for each individual slice by cumulative planimetry using computerized Image‐Pro Plus 6.0 (Media Cybernetics Inc., Silver Spring, MD, USA).

### Measurement of serum enzymatic activity/level

After hemodynamic studies, blood samples were rapidly collected and serum was obtained by centrifugation at 4500 × g for 6 min. Serum levels of creatine kinase myocardium (CK‐MB) and lactate dehydrogenase (LDH) were detected with biochemical detecting system (AU2700; Olympus Melville, NY, USA). Serum cardiac troponin I (cTnI), citrate synthase (CS) and cytochrome C oxidase (CCO) activity were measured using a rat ELISA kit (Beyotime Biotech, Haimen, China) according to the manufacturer's instructions.

### TUNEL staining

Tissue cryosections were stained using the TUNEL system (Promega, Madison, WI, USA) according to the manufacturer's protocol, and staining was observed under fluorescence microscopy (TE‐2000U; Nikon, Tokyo, Japan). The level of apoptotic cardiomyocytes was shown as a percentage of the number of TUNEL‐positive cells to the number of total cells.

### Determination of ATP content

Myocardial tissue ATP content was determined by an Enhanced ATP Assay Kit (Beyotime, China) according to the manufacturer's instructions, and the results are shown in arbitrary units.

### Preparation of mitochondrial fractions

Isolation of mitochondria was performed with the Mitochondria Fractionation Kit (Beyotime Biotech, Haimen, China) according to the manufacturer's protocol. Briefly, fresh cardiac tissue was mixed with a mitochondria extraction reagent and stirred in a homogenizer and the suspension was centrifuged at 1000 × g for 5 min. (4°C); the supernatant obtained was centrifuged at 3500 × g for 10 min. (4°C) and the precipitate contained the mitochondrial fraction.

### Mitochondrial membrane potential determination

Mitochondrial membrane potential (ψ_m_) was measured using the JC‐1 assay kit (Beyotime Biotech) according to the manufacturer's instructions. Fresh isolated mitochondria were incubated with an equal volume of JC‐1 staining solution (10 mg/ml) for 20 min. at 37°C in the dark and rinsed twice with buffer. JC‐1 fluorescence was measured by a fluorescence microscope under single excitation (488 nm) and dual emission (shift from 530 to 590 nm). The ratio of green and red fluorescent intensities indicated changes in mitochondrial membrane potential.

### Assessment of MPTP

Mitochondrial permeability transition pore opening was assessed using a commercially available kit (Genmed Scientifics Inc., Boston, MA, USA) according to the manufacturer's instructions. Briefly, purified mitochondria (10 mg/ml, 20 μl) were transferred to 96‐well plates and incubated with pre‐warmed Reagent A. After 1 min., inducing medium (Reagent B, including CaCl_2_) was added and absorbance at 540 nm was measured using a multi‐mode microplate reader (Awareness Technology, Palm City, FL, USA), with decrease in absorbance indicating transition in mitochondrial permeability.

### Transmission electron microscopy

Fresh LV tissue isolated was fixed with 2.5% glutaraldehyde in 0.1 M phosphate buffer for 2 hrs at 4°C. Following fixation with 1% osmium tetroxide in 0.1 M phosphate buffer, the tissue was dehydrated with a graded series of ethanol to 100% and infiltrated with propylene oxide to embedding media (Epon 812 resin). Ultrathin sections were cut with an ultramicrotome, post‐stained with uranyl acetate and lead citrate, and then viewed by transmission electron microscopy (TEM; H‐7650; Hitachi, Tokyo, Japan). Digital images were analysed using Image J to manually generate masks of mitochondrial contours that were then used for the calculation of mitochondrial area, perimeter, maximum diameter and total mitochondrial number.

### Examination of mitochondrial size and complexity

Mitochondrial size and complexity were evaluated using flow cytometry 30. MitoTracker Green (Beyotime), which passively accumulates in the lipid environment of intact mitochondria, was used to selectively stain intact mitochondria. Debris in the samples did not have a lipid environment and was excluded to obtain an accurate gating (R1) of the mitochondria. After establishing gating parameters, gated events (10,000/sample) were analysed using the forward scatter detector (FSC) and side scatter detector (SSC), and the results are shown in an FSC *versus* SSC density plot. The geometric mean (arbitrary units) representing FSC (logarithmic scale) was defined to depict size, whereas data from SSC (logarithmic scale) were considered an indicator of complexity.

### Western blot

Cardiac tissue proteins were extracted with a protease inhibitor containing lysis buffer, sample proteins were resolved on SDS‐PAGE, and then transferred to polyvinylidene fluoride membranes to be probed with primary antibody overnight and then washed before incubation with horseradish peroxidase‐linked secondary antibody for 30 min. at room temperature. Bands were visualized with ECL‐Plus reagent (Millipore, Billerica, MA, USA) and detected by a ChemiDoc‐It Imaging System (Upland, CA, USA).

### Statistical analysis

Results are presented as the mean ± S.E.M. Data analysis was processed with one‐way anova followed by a Tukey *post hoc* test or Student's *t*‐test. *P* < 0.05 was considered indicative of statistical significance. Statistical analyses were performed using GraphPad Prism Version 5.01 (GraphPad Software, La Jolla, CA, USA).

## Results

### VNS treatment attenuates damage in ISO‐induced myocardial ischaemia

**Figure 2:**
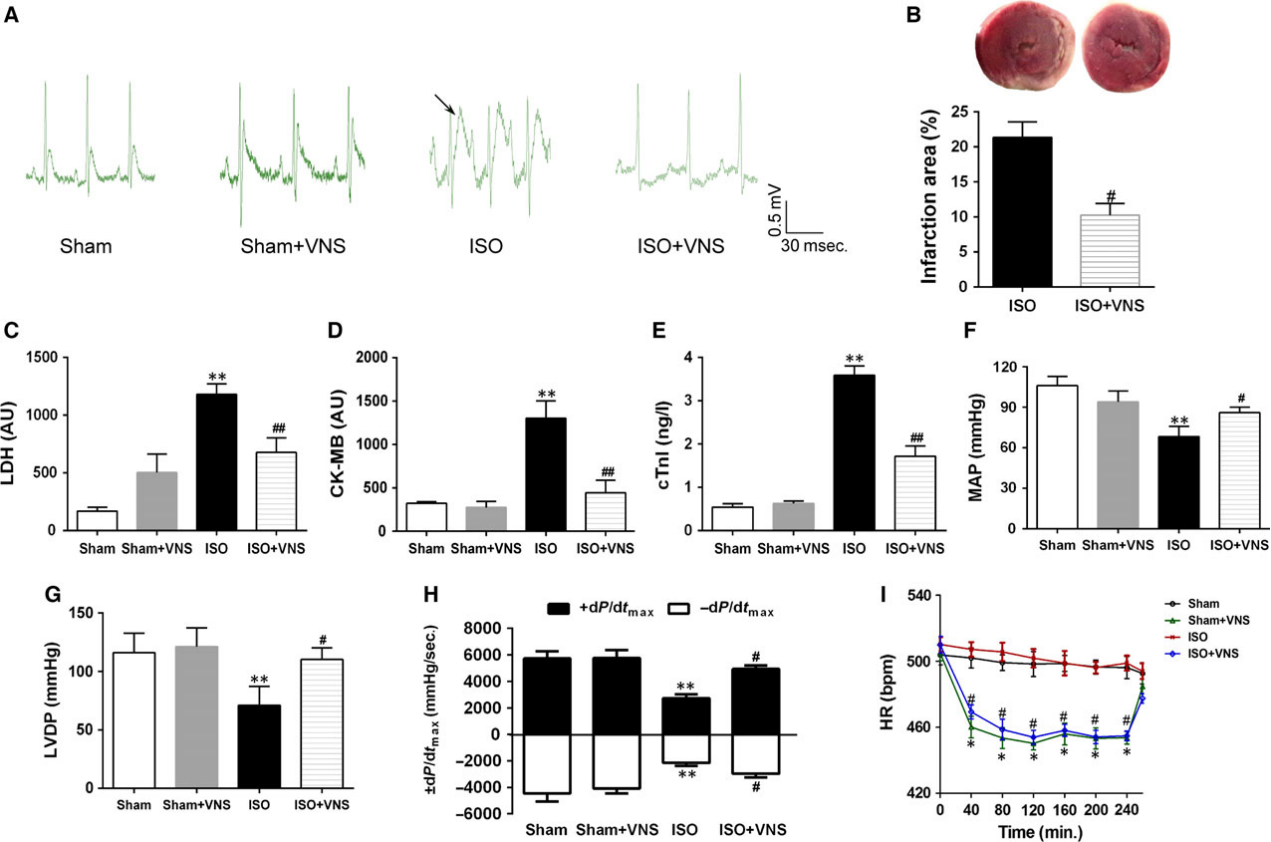
VNS treatment protects cardiac function against ISO‐induced myocardial damage in a rat model. (**A**) Electrocardiogram. Arrow denotes elevated ST‐segment in the ISO‐treated group. (**B**) Measurement of myocardial infarct size. (**C**–**E**) Detection of myocardial enzymes in serum. (**F**–**H**) Haemodynamics analysis. (**I**) HR during stimulation period. LDH, lactate dehydrogenase; CK‐MB, creatine kinase‐MB; cTnI, cardiac troponin‐I; MAP, mean arterial pressure; LVDP, LV developed pressure; ±dP/dt_max_, maximal rate of the increase/decrease in LV pressure; HR, heart rate. *n* = 6, **P < 0.01 *versus* Sham; ^#^ *P* < 0.05 *versus* ISO; ^##^ *P* < 0.01 *versus* ISO.

To verify whether ISO treatment induced myocardial ischaemia, electrocardiographic parameters and serum myocardial enzymes were measured. In the ISO group, a marked elevation of the ST‐segment and increased levels of diagnostic marker enzymes (LDH, CK‐MB and cTnI) was evident, compared to the sham group, clearly suggesting that ISO treatment caused myocardial ischaemia (Fig. 2A and C–E). Vagal nerve stimulation in ISO‐administered rats significantly decreased ST‐segment elevation, infarct size and levels of serum myocardial enzymes, compared with the ISO group (*P* < 0.05; Fig. 2A–E). Otherwise, compared with the sham group, ISO reduced hemodynamic parameters, mean arterial pressure (MAP), LV developed pressure (LVDP) and ±dP/dt_max_, whereas the effect was partially ameliorated after VNS (*P* < 0.05; Fig. 2F–H). These data indicate that VNS treatment improves cardiac function and reduces myocardial infarct size. Furthermore, to ensure effectiveness of VNS, HR during the stimulation period was monitored. As shown in Figure 2I, a 10% HR reduction was consistent during VNS treatment, and after cessation of VNS, HR showed an upward trend towards the baseline level, which indicated that the heart‐rate reduction was achieved by VNS.

### VNS decreases cardiomyocyte apoptosis induced by ISO

**Figure 3:**
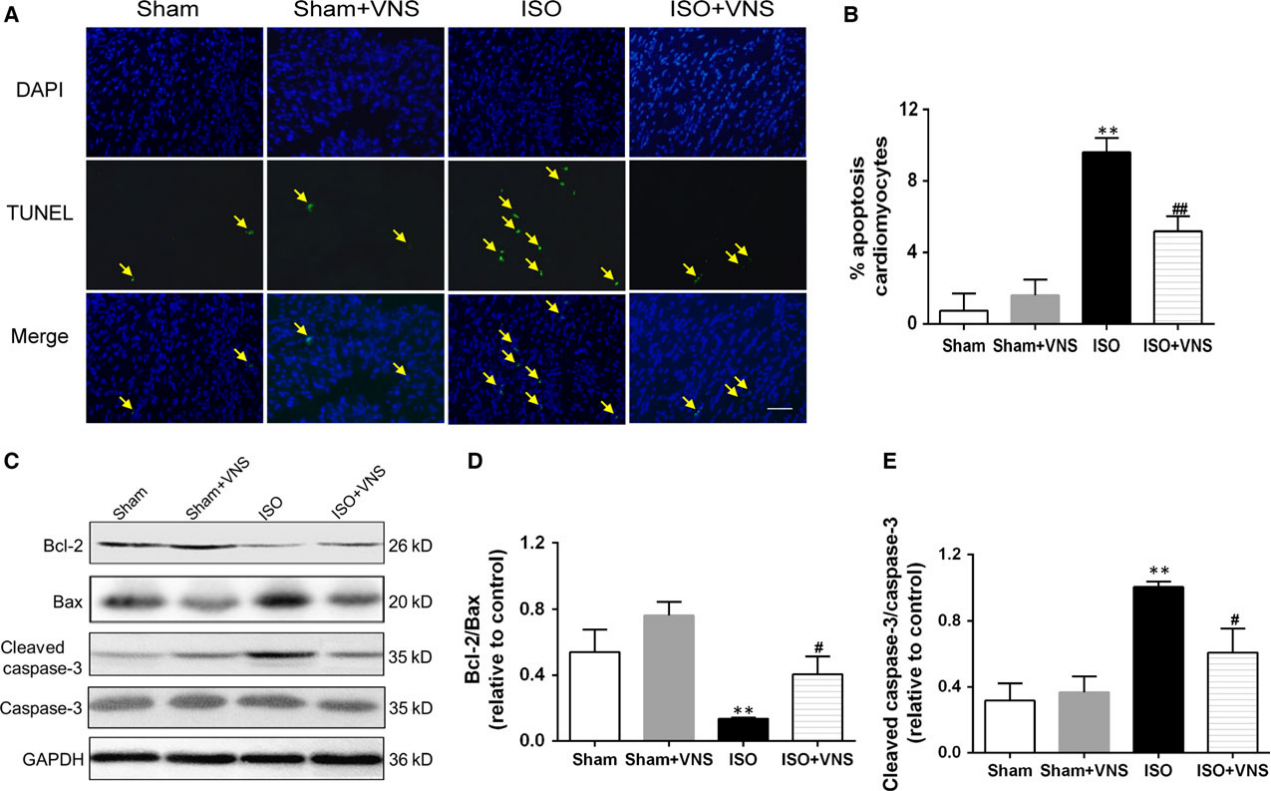
VNS treatment ameliorates ISO‐induced myocardial apoptosis. (**A**) Fluorescence microscopy images indicating TUNEL (green) and cell nuclei (blue) staining. Arrows indicate TUNEL‐positive cells, scale bar = 100 μm. (**B**) Quantification of apoptotic cardiomyocytes. (**C**) Representative immunoblots and (**D** and **E**) Western blot analysis of Bcl‐2/Bax and cleaved caspase‐3/caspase‐3 expression changes. *n* = 6, **P < 0.01 *versus* Sham; ^#^ *P* < 0.05 *versus* ISO; ^##^ *P* < 0.01 *versus* ISO.

To elucidate whether VNS plays a positive cardioprotective role, cardiomyocyte apoptosis was measured using the TUNEL assay, the Bcl‐2/Bax ratio, and the level of cleaved caspase‐3. Isoproterenol significantly induced myocardial apoptosis compared with sham treatment, indicated by an increase in the number of TUNEL‐positive cells, decrease in the Bcl‐2/Bax ratio and an increase in the level of cleaved‐caspase‐3. Vagal nerve stimulation markedly ameliorated the amount of ISO‐induced TUNEL‐positive cells (*P* < 0.01; Fig. 3A and B), enhanced the expression of Bcl‐2, an anti‐apoptotic factor, and raised the Bcl‐2/Bax ratio (*P* < 0.05; Fig. 3C and D), while it decreased the level of cleaved caspase‐3 and downregulated the cleaved caspase‐3 ratio (*P* < 0.05; Fig. 3C and E). This suggests that VNS treatment reduces ISO‐induced apoptosis of cardiomyocytes.

### VNS regulates the expression of mitochondrial dynamics proteins in ISO‐induced myocardial ischaemia

**Figure 4:**
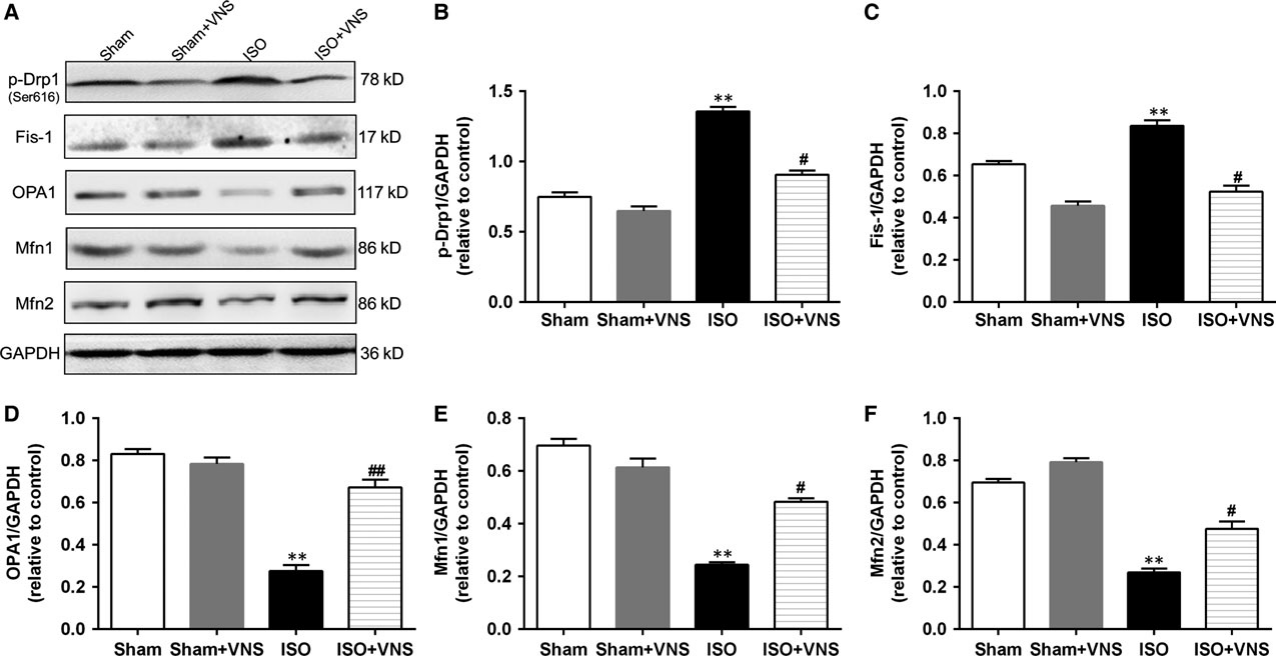
VNS treatment partially restores the expression of ISO‐induced cardiac mitochondrial dynamics proteins. (**A**) Representative immunoblots and (**B**–**F**) Western blot analysis of the mitochondrial dynamics protein expression of p‐Drp1, Fis‐1, OPA1, Mfn1, and Mfn2. *n* = 6, **P < 0.01 *versus* Sham; ^#^ *P* < 0.05 *versus* ISO; ^##^ *P* < 0.01 *versus* ISO.

To further study whether mitochondria is injured in ISO‐induced myocyte, we examined the major proteins controlling mitochondrial morphology by examining the expression of Fis‐1 and Drp1 (proteins which regulate fission events), OPA1 (which regulates mitochondrial inner membrane fusion), and Mfn1/2 (which controls outer membrane fusion). Phosphorylation of Drp1 at Ser616 promotes translocation of Drp1 from the cytosol to the mitochondrial membrane to mediate fission 31. Compared with the sham group, expression of Fis‐1 and p‐Drp1 increased and of Mfn1/2 and OPA1 decreased in the ISO group. This change manifested as enhanced fission and weakened fusion in the mitochondria, with resultant mitochondrial dysfunction and morphologic changes. Vagal nerve stimulation treatment not only suppressed p‐Drp1 and Fis‐1 expression but also restored Mfn1/2 and OPA1 levels compared to the ISO group (*P* < 0.05; Fig. 4), suggesting that VNS alleviates the shift in balance between fission and fusion following myocardial ischaemia.

### VNS suppresses ISO‐induced cardiac mitochondrial dysfunction and morphological abnormality

**Figure 5:**
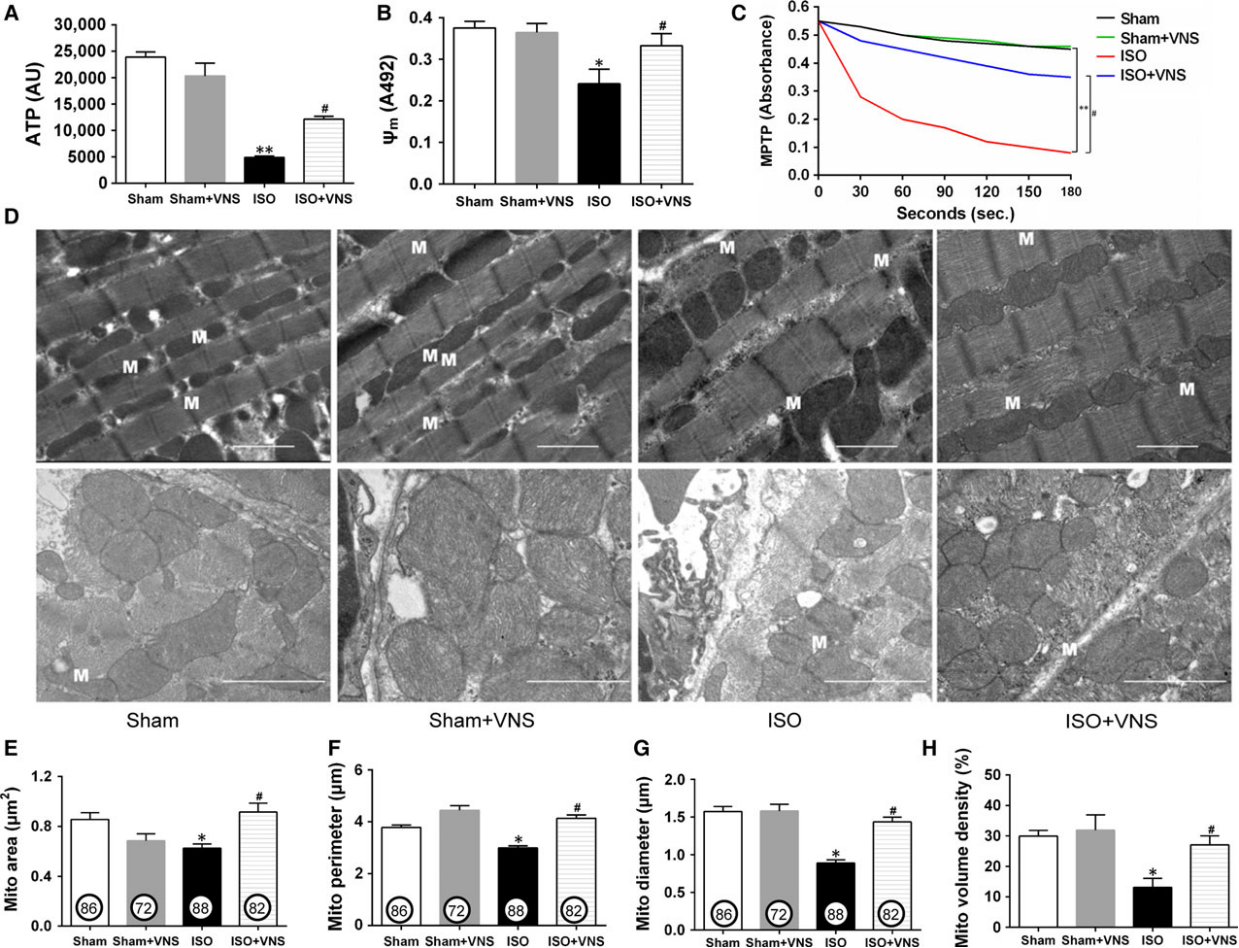
VNS suppresses ISO‐induced mitochondrial dysfunction and morphological damage. (**A**–**C**) Levels of myocardial ATP content (**A**), mitochondrial membrane potential (**B**), and mitochondrial permeability transition pore opening (**C**). (**D**) Transverse (top) and longitudinal (bottom) sections of mitochondria in cardiomyocytes from each experimental rat. M: mitochondrion, scale bar = 2 μm. (**E**–**G**) Area, perimeter and diameter of each individual mitochondrion estimated from electron microscopic image. Numbers in circles represent the number of fields analysed per group. (**H**) Mitochondrial volume density as measured by the grid analysis and expressed as a percentage. *n* = 6, *P < 0.05 *versus* Sham; **P < 0.01 *versus* Sham; ^#^ *P* < 0.05 *versus* ISO.

**Figure 6:**
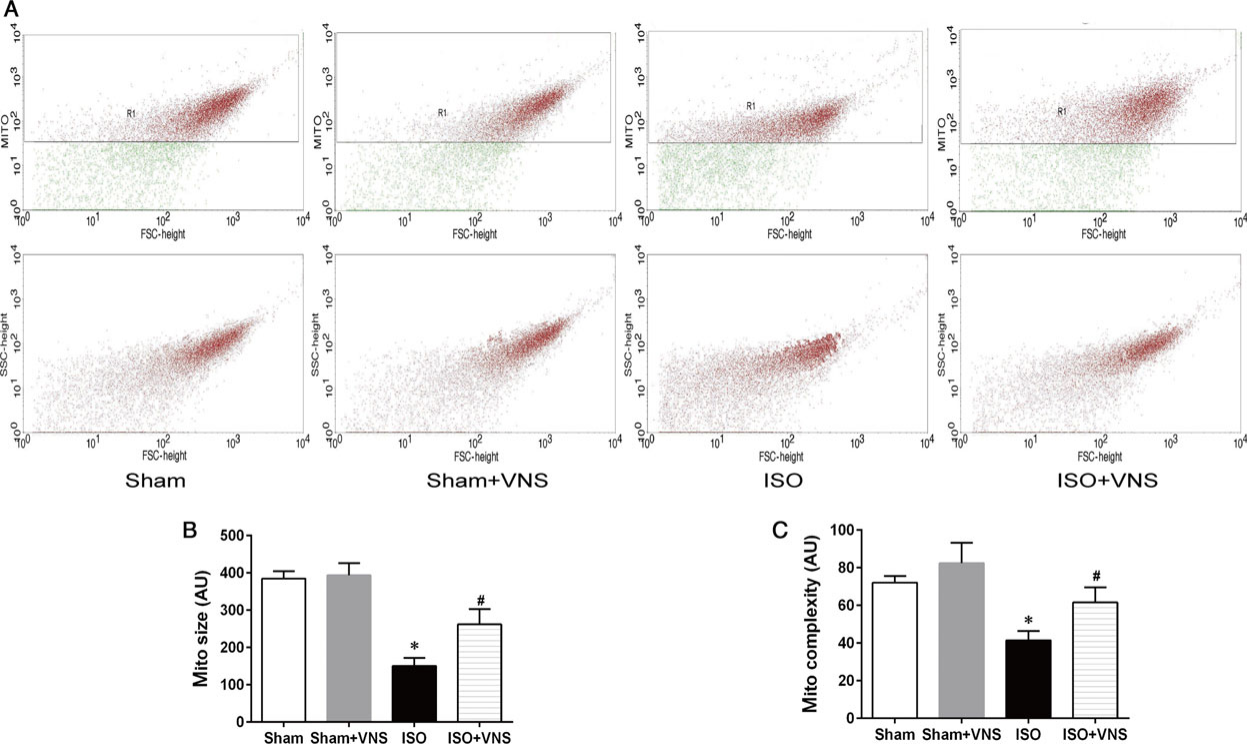
VNS attenuates ISO‐induced mitochondrial size and integrity. Relative size and internal complexity of cardiac mitochondria isolated from each experimental group were determined by flow cytometry. (**A**) Intact mitochondria are in red and debris and noise are shown in green (top) in representative gated‐density plots (R1 zoom) from each group of rats indicating size (FSC) *versus* internal complexity (SSC) of isolated mitochondria (bottom). (**B**) Analysis of isolated cardiac mitochondrial size in each experimental group. (**C**) Analysis of isolated cardiac mitochondria complexity in each experimental group. FSC, forward scatter; SSC, side scatter; AU, arbitrary units. *n* = 6, *P < 0.05 *versus* Sham; ^#^ *P* < 0.05 *versus* ISO.

Mitochondrial ATP content, membrane potential and MPTP opening were determined to elucidate the effects of VNS on mitochondrial function. In the ISO group, the ATP content and mitochondrial membrane potential decreased dramatically with MPTP opening, as compared to the sham group, and this was reversed by VNS (*P* < 0.05; Fig. 5A–C). Moreover, cardiac mitochondrial ultrastructure and morphology were evaluated by TEM. In the sham group, mitochondria had an even, elongated shape and were strictly aligned between myofibrils, whereas in the ISO‐induced cardiomyocyte, mitochondrial arrangement was irregular, with clusters of mitochondrial fragments and high diversity in shape and size—an effect partially inhibited by VNS (Fig. 5D, top). We further quantified these morphological changes (Fig. 5D, bottom). Morphological analysis indicated ISO treatment induced a significant reduction in mitochondrial area, perimeter, diameter, and density, compared with the sham group that reverted to baseline after VNS (*P* < 0.05; Fig. 5E–H). To validate these findings, isolated mitochondrial morphology was analysed using flow cytometry. Mitochondrial size (FSC) and internal complexity (SSC) in the ISO group decreased significantly when compared with the sham group—an effect that was partially ameliorated after VNS during myocardial ischaemia (*P* < 0.05; Fig. 6). These morphological observations indicate VNS treatment can alleviate myocardial organelle, particularly mitochondrial, damage induced by myocardial ischaemia. Taken together, these data indicate VNS mitigates ISO‐induced cardiac mitochondrial dysfunction and morphological abnormality.

### VNS activates AMPK through preferred phosphorylation of CaMKKβ, instead of LKB1, in ISO‐induced myocardial ischaemia

**Figure 7:**
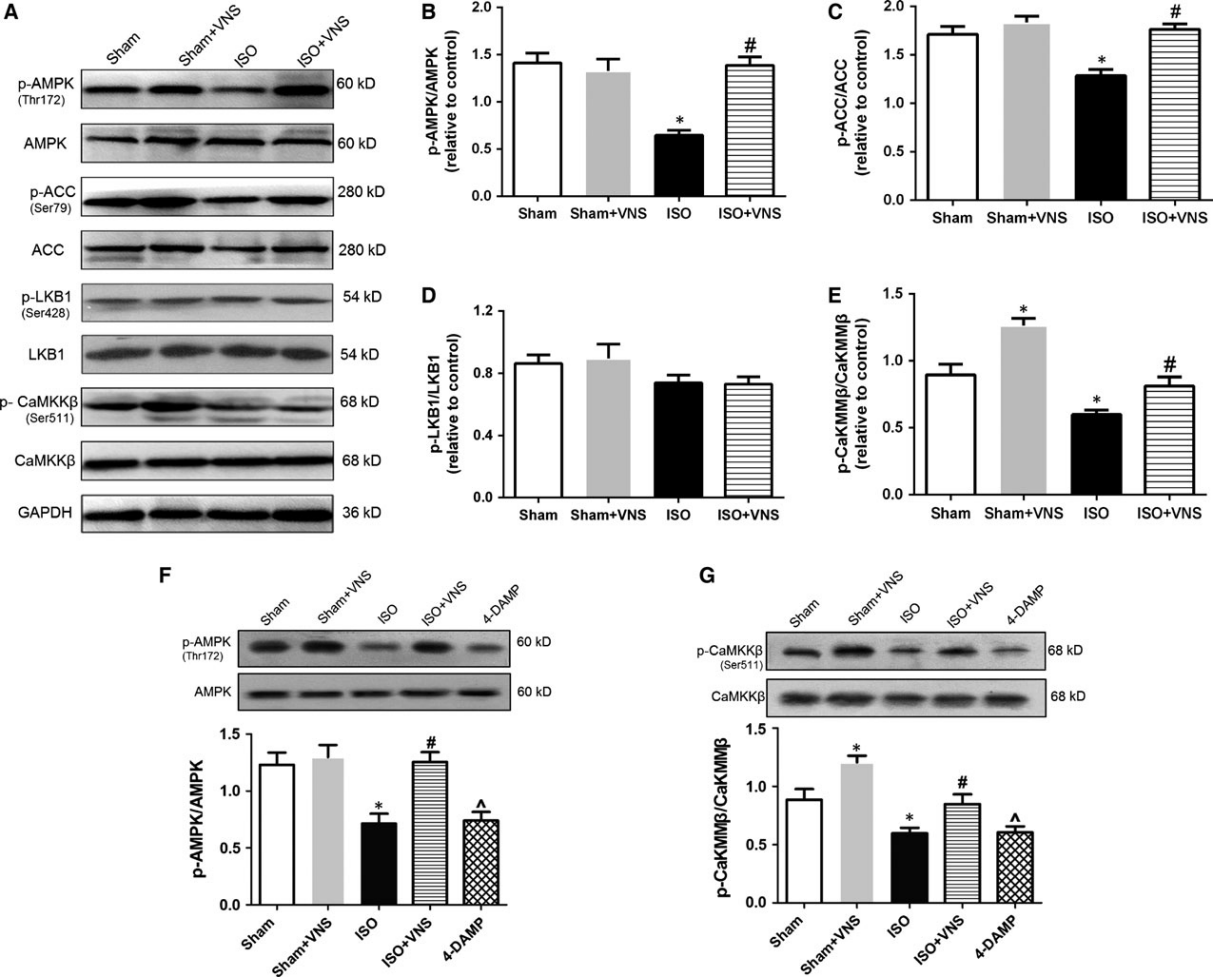
VNS activates AMPK *via* CaMKKβ, rather than LKB1, phosphorylation. (**A**) Representative Western blot showing protein expression of p‐AMPK, AMPK, p‐ACC, ACC, p‐LKB, LKB, p‐CaMKKβ, CaMKKβ, and GAPDH. (**B**) Quantitative analysis of AMPK phosphorylation. (**C**–**E**) Quantitative analysis of ACC phosphorylation (a substrate protein of AMPK) and LKB1 and CaMKKβ phosphorylation (upstream kinases of AMPK). (**F** and **G**) Western blot analysis of p‐AMPK/AMPK and p‐CaMKKβ/CaMKKβ. *n* = 6, *P < 0.05 *versus* Sham; ^#^ *P* < 0.05 *versus* ISO ; ^*P* < 0.05 *versus* ISO.

To the best of our knowledge, AMPK activation can protect the heart against myocardial IRI through regulation of mitochondrial function 26. Therefore, we aimed to determine whether the AMPK pathway is involved in VNS‐mediated regulation of mitochondrial dynamics. First, we examined the phosphorylation of AMPK and acetyl‐CoA carboxylase (ACC, a substrate of AMPK), and found these were reduced in the ISO group, compared with the sham group, but were restored by VNS treatment (*P* < 0.05; Fig. 7B and C). Second, we analysed two kinases predicted to be upstream of AMPK—liver kinase B1 (LKB1) and Ca^2+^/calmodulin‐dependent kinase kinase β (CaMKKβ). As shown in Figure 7D and E, although there was no significant difference in the phosphorylation of LKB1 between any of the four groups, a significant decrease in CaMKKβ phosphorylation was evident in the ISO group. Interestingly, VNS caused CaMKKβ phosphorylation increase in both the VNS alone and the VNS+ISO group (*P* < 0.05), indicating that VNS may potentially activate AMPK through phosphorylation of CaMKKβ rather than LKB1.

Furthermore, 4‐DAMP (a selective M_3_R antagonist) was used to determine the mechanism by which VNS activates CaMKKβ and its downstream kinases, because other research reported Gq receptors (activated by M_1_R, M_3_R and M_5_R) to be upstream for CaMKKβ/AMPK signalling 32, with choline—a precursor and metabolite of ACh—demonstrated to produce beneficial effects on the heart *via* cardiac M_3_R activation 33, 34. As shown in Figure 7F and G, inhibition of M_3_R decreased expression of p‐AMPK and p‐CaMKKβ, suggesting a possible role of M_3_R in the activation of the CaMKKβ/AMPK pathway by VNS.

### Inhibition of AMPK or M_3_R compromises VNS‐induced protective effects on mitochondrial dynamics and function

**Figure 8:**
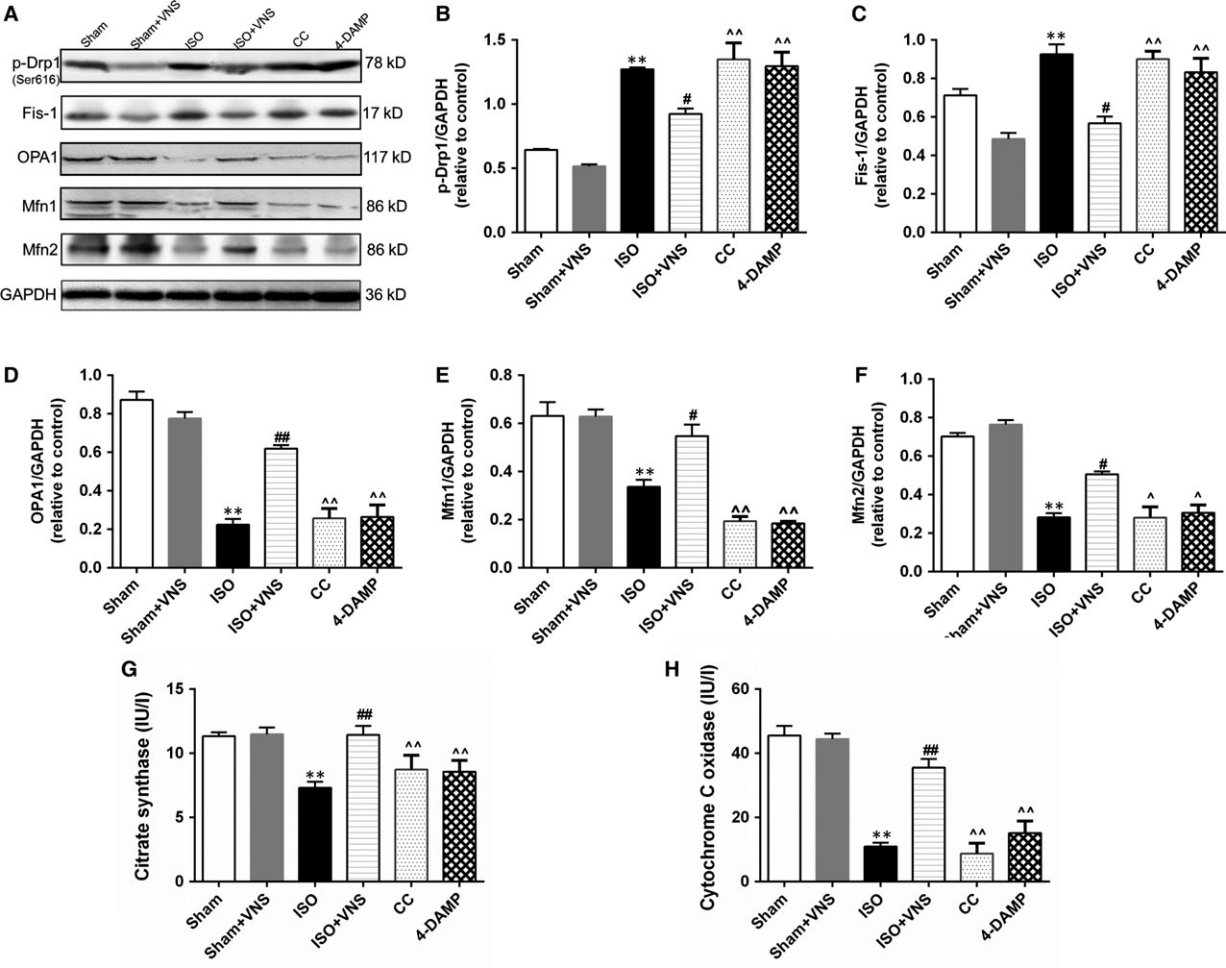
Inhibition of AMPK and M_3_R reverses the protective effect of VNS on mitochondrial dynamics and function. (**A**–**F**) Representative western blot and quantitative analysis measuring the expression of p‐Drp1, Fis‐1, OPA1, Mfn1, Mfn2 and GAPDH. (**G** and **H**) Detection of mitochondrial citrate synthase (CS) and cytochrome C oxidase (CCO) activity. *n* = 6, **P < 0.01 *versus* Sham; ^#^ *P* < 0.05 *versus* ISO; ^##^ *P* < 0.01 *versus* ISO; ^*P* < 0.05 *versus* ISO+VNS; ^^*P* < 0.01 *versus* ISO+VNS.

To further determine the role of AMPK and related pathways in the regulation of mitochondrial dynamics following VNS treatment, we used Compound C (an AMPK inhibitor) and 4‐DAMP. Compared with the ISO+VNS group, both AMPK and M_3_R inhibition increased p‐Drp1 and Fis‐1 expression, whereas expression of OPA1 and Mfn1/2 were significantly reduced (*P* < 0.05; Fig. 8A–F), indicating AMPK may function as an essential link between VNS treatment and mitochondrial dynamics and M_3_R acts as a bridge for VNS to activate the CaMKKβ/AMPK pathway. Moreover, we determined the effect of AMPK and M_3_R inhibition on mitochondrial function. Compared with the ISO+VNS group, AMPK and M_3_R inhibition led to a reduction in mitochondrial CS and CCO activity (*P* < 0.01; Fig. 8G and H), corroborating the hypothesis that M_3_R and AMPK are involved in the regulation of mitochondrial dynamics and function that is induced by VNS during myocardial ischaemia.

## Discussion

**Figure 9:**
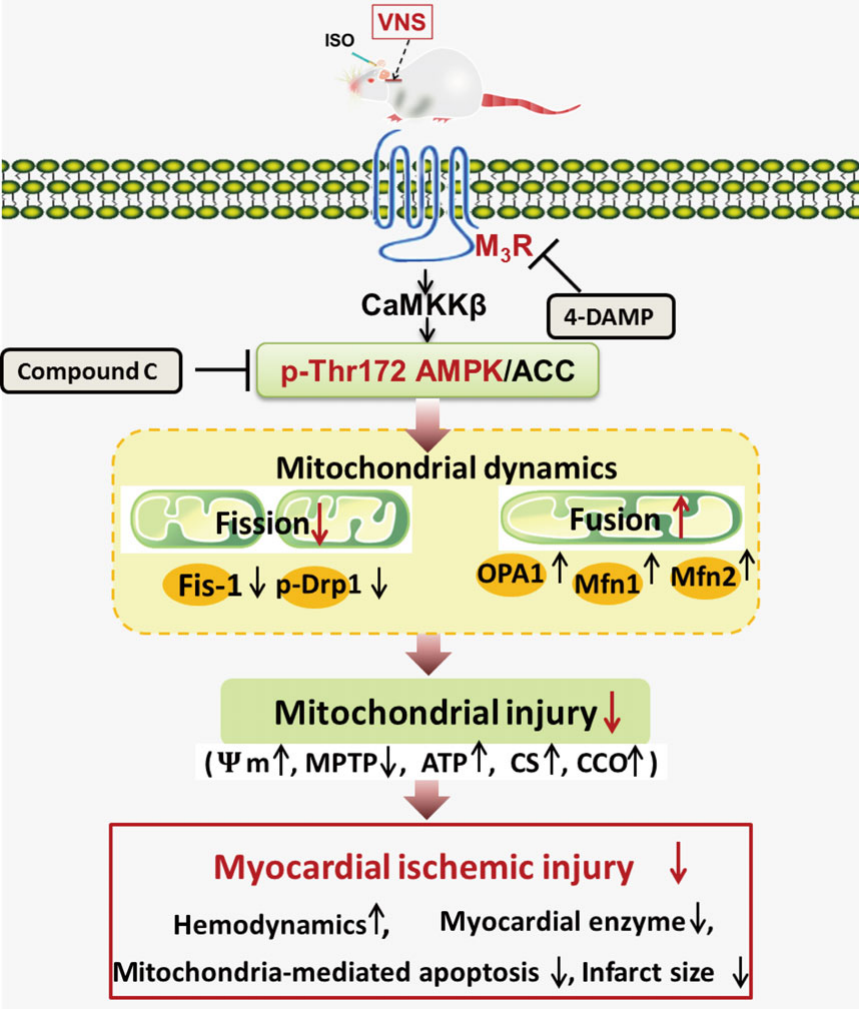
Schematic illustration of VNS regulation of mitochondrial fission and fusion through an M_3_R/CaMKKβ/AMPK pathway in rats with ISO‐induced myocardial ischaemia. AMPK, AMP‐activated protein kinase; AAC, acetyl‐CoA carboxylase; CaMKKβ, Ca^2+^/calmodulin‐dependent protein kinase kinase β; CS, citrate synthase; CCO, cytochrome C oxidase; Drp1, dynamin‐related peptide1; Fis‐1, mitochondrial fission protein1; ISO, isoproterenol; MPTP, mitochondrial permeability transition pore; M_3_R, subtype 3 of muscarinic acetylcholine receptor; Mfn1, mitofusin1; Mfn2, mitofusin2; OPA1, optic atrophy‐1; VNS, vagal nerve stimulation; 4‐DAMP, 4‐diphenylacetoxy‐*N*‐methypiperidine methiodide; ψ_m_, mitochondrial membrane potential.

Mitochondrial damage contributes to cardiac dysfunction and cardiomyocyte injury *via* loss of metabolic capacity as well as production and release of toxic products during myocardial ischaemia. This study focused on changes in mitochondrial dynamics and the signalling pathways involved during myocardial ischaemia, with or without VNS treatment. Our results found that: (*i*) ISO treatment disrupts myocardial mitochondrial dynamics, as evidenced by downregulation of mitochondrial fusion proteins (OPA1 and Mfn1/2) and up‐regulation of mitochondrial fission proteins (p‐Drp1 and Fis‐1) and a large number of mitochondrial fragments in rat heart; (*ii*) VNS not only alleviated myocardial infarct size, reduced apoptosis and improved cardiac function but also up‐regulated expression of mitochondrial fusion proteins (OPA1 and Mfn1/2) and downregulated the levels of mitochondrial fission proteins (p‐Drp1 and Fis‐1), thereby reversing the damaging effects of ISO‐induced myocardial ischaemia, subsequently leading to improved mitochondrial function and morphology evidenced by increased ATP content and mitochondrial membrane potential and a reduction in MPTP opening and (*iii*) importantly, VNS activated AMPK through CaMKKβ, but not LKB1, phosphorylation. Treatment with either the M_3_R blocker 4‐DAMP or AMPK inhibitor Compound C mitigated the positive effects of VNS on mitochondrial dynamic protein expression together with CS and CCO activities. Taken together, these novel findings suggest VNS elicits an improvement in mitochondrial dynamics, possibly through an M_3_R/CaMKKβ/AMPK signalling pathway during myocardial ischaemia (Fig. 9).

Three main methods exist for the development of a MI model in rats: coronary artery ligation, an electrocautery technique applied to the epicardial surface, and administration of ISO 35. Although left coronary artery ligation is most frequently used to induce acute myocardial damage in rat, this surgical procedure has disadvantages of a high mortality rate and a large variation in infarct size 36, 37. The extent of cardiac damage produced by the electrical method, consisting of overlapping burns, is not consistent among laboratories, limiting the reproducibility of the results obtained with this procedure 36. Pharmacological induction of heart damage is achieved by treatment with the β‐adrenergic receptor agonist ISO 38, which is a classic and easy method to induce myocardial ischaemia. In addition, it has reported that ISO has deleterious cardiac effects in rat, including necrosis, apoptosis, mitochondrial alterations, hypertrophy, fibrosis, oxidative damage and inflammatory cell infiltration, which is similar to that in the infarcted human heart 3, 39, 40. Importantly, ISO induction could imitate sympathetic overexcited state in myocardial ischaemia, which is in line with the recent found that imbalanced autonomic nervous system (excessive sympathetic activity and reduced vagal activity) exists in most of the myocardial ischaemic patients 41. In our study, 25 mg/kg ISO induced myocardial injury, demonstrated by elevation of the ST‐segment and levels of LDH, CK‐MB and cTnI. Furthermore, ISO reduced hemodynamic parameters (MAP, LVDP, and ±dP/dt_max_) and increased the infarct area. Taken together, ISO induces an acute MI and, additionally, induced mitochondrial dysfunction—including impaired oxidative metabolism, calcium mishandling 42, decreased mitochondrial bioenergetics and enhanced oxidative stress 43, but the effects on mitochondrial dynamics were rare.

Mitochondria are dynamic organs and undergo fusion and division that generate interconnected mitochondrial networks facilitating physiological cell adaptation. Generally, mitochondrial outer and inner membrane fusion events are, respectively, mediated by Mfn1/2 and OPA1. Fused mitochondria are required for transmission of membrane potential to dissipate metabolic energy and to exchange mtDNA products in heteroplasmic cells to defend against ageing 44, 45. In contrast, phosphorylation of Drp1 on Ser616 promotes mitochondrial fission 31. Fis‐1, as a crucial receptor, assists Drp1 to complete the fission event 46. Fission is crucial for mitochondrial inheritance through growth and division, cytochrome C release to promote apoptosis, and turnover of damaged organelles by mitophagy 47, 48. Thus, it is important to define the role of mitochondrial dynamics in cardiovascular diseases. Lam *et al*. reported that ISO accelerated turnover of proteins mediating mitochondrial dynamics, such as Miro1/2, LONP, PHB and most respiratory chain components; however, Mfn1/2 and Fis‐1 states remained unchanged 49. Our results showed ISO‐induced myocardial ischaemia was followed by a slew of mitochondrial debris in cardiac tissues—an indication of a defect in mitochondrial dynamics in hearts subjected to ischaemia. This result concurs with the study performed in HL‐1 cardiomyocytes 50. Moreover, in the ISO group, the expression of mitochondrial fission proteins p‐Drp1 and Fis‐1 was significantly enhanced whereas that of fusion proteins OPA1 and Mfn1/2 was weakened when compared to the sham group. Thus, it is reasonable to consider that repair of imbalanced mitochondrial dynamics could benefit mitochondrial and cardiac function and might represent a potential strategic target for treatment in myocardial ischaemia.

Emerging evidence supports the idea that improved vagal tone has markedly protective effects on attenuating cardiac mitochondrial ROS generation, decreasing mitochondrial swelling, cytochrome C release, inhibiting MPTP opening and increasing ATP production 22, 51, 52. So, what does VNS modulate to induce these positive effects on mitochondrial function? This study found that VNS rectified the ISO‐induced turbulence in mitochondrial dynamics—it weakened the expression of p‐Drp1 and Fis‐1 and up‐regulated OPA1 and Mfn1/2, subsequently decreasing the amount of mitochondrial fragments and increasing the elongated mitochondrial network. Furthermore, not only were the mitochondrial dynamics proteins involved in mitochondrial morphology but they were also involved in regulating mitochondrial function. Inhibition of Drp1 has been shown to suppress mitochondria‐mediated apoptosis and Bax facilitates Drp1 translocation to the mitochondrial membrane to promote fission events 53, 54. On the other hand, OPA1 has been shown to play a critical role in maintaining cristae junctions, and disruption of OPA1 results in changes to cristae morphology and impaired mitochondrial metabolic ability 55, 56. After VNS rectified mitochondrial dynamics, mitochondrial dysfunction was also reversed in this study. Vagal nerve stimulation decreased p‐Drp1 level, caused recovery of mitochondrial membrane potential, decreased MPTP opening, and reduced the number of injured mitochondrial‐mediated myocardial cells undergoing apoptosis. Moreover, treatment with VNS promoted OPA1 expression and protected mitochondrial ultrastructure and metabolism, including by enhancing the activity of mitochondrial metabolic enzymes (e.g. CS and CCO) and ATP content. Therefore, we theorize VNS regulates mitochondrial dynamics, thereby preventing damage induced by myocardial ischaemia.

AMPK (a conserved energy sensor) plays an important role in regulating cell survival and death in response to pathological stress, including ER, oxidative and osmotic stress 57, 58. This crucial function of AMPK was demonstrated by a study which demonstrated that treatment with AMPK activators increased cell viability 59. Moreover, our previous research demonstrated that AMPK activation played an important role in VNS or ACh ability to protect from mitochondrial biogenesis and antioxidative stress, and provide autophagic cytoprotection during myocardial ischaemia 13, 60, 61. More interestingly, another study demonstrated that AMPK activation prevented mitochondrial fission by decreasing Drp1 and Fis‐1 levels in high glucose‐induced endothelial apoptosis 62. In this study, we showed that VNS treatment activated AMPK and ACC (the substrate of AMPK) in ISO‐induced myocardial damage. Furthermore, inhibition of AMPK by Compound C mitigated the protective effect of VNS, suggesting that AMPK was indeed involved in VNS‐mediated protection of mitochondrial dynamics and function. In mammalian cells, CaMKKβ and LKB1 are thought to be the two major upstream kinases of AMPK 63, 64. This study found that VNS activation of AMPK was accompanied by increased CaMKKβ, but not LKB1, phosphorylation, which may be explained by the change in cytosolic Ca^2+^ level 65, moreover, it should be noted that CaMKKβ is directly regulated by Ca^2+^. Our previous study demonstrated that ACh expression inhibited hypoxia/reoxygenation‐induced intracellular Ca^2+^ overload to prevent mitochondrial damage in vascular endothelial cells 66. Furthermore, Mungai *et al*. demonstrated that moderate hypoxia‐induced AMPK activation occurred through a calcium‐mediated pathway and was abolished by knockdown of CaMKKβ, but not LKB1, which suggested that a Ca^2+^/CaMKKβ/AMPK pathway could enhance the ability of mitochondria to protect cells against a more severe insult 67. We hypothesize that, in response to ISO‐induced myocardial ischaemia, VNS stimulates intercellular Ca^2+^ release to protect mitochondria and VNS activates a CaMKKβ/AMPK pathway to alleviate myocardial ischaemia through mitochondrial protection.

Activation of the CaMKKβ/AMPK pathway is usually induced by Gq‐coupled receptors, including M_1,3,5_R 32, all of which are involved in the regulation of energy metabolism 68, 69. Further study showed that M_3_R plays a critical role in glucose homoeostasis 68, 70. Some research reported that M_3_R activation by choline, an ACh precursor and metabolite, has a protective effect on both cardiac and vascular endothelial cells by enhanced phosphorylation of Connexin‐43, Ca^2+^/calmodulin‐dependent protein kinase II (CaMK II), and endogenous antioxidant capacity, and diminished Ca^2+^ overload 33, 34, 71, 72, 73. Moreover, M_3_R activation decreased Bcl‐2 expression and cytochrome C release and attenuated mitochondria‐mediated apoptosis 22, 72. In this work, the results showed that M_3_R inhibition decreased p‐AMPK and p‐CaMKKβ expression and blocked the beneficial effects of VNS on mitochondrial dynamics and function during myocardial ischaemia, suggesting the possible involvement of M_3_R in the activation of the CaMKKβ/AMPK pathway by VNS. Further studies are needed to clarify the role of M_3_R in the cardioprotective action of VNS.

In summary, our study offers salient evidence that VNS protected mitochondrial fusion and fission, and functions against ISO‐induced myocardial ischaemia. Notably, we indicated that VNS‐mediated mitochondrial protection possibly acts through activation of M_3_R/CaMKKβ/AMPK pathway. Our findings provide new insights into the mechanism underlying VNS‐mediated cardioprotection, indicating that protection of mitochondria through VNS treatment could be a potential strategy to prevent myocardial ischaemia.

## Conflicts of interest

The authors declare no conflict of interest.
